# Primary Yolk Sac Tumor of the Liver: A Rare Cause of Bile Duct Obstruction

**DOI:** 10.1155/2024/5549996

**Published:** 2024-02-27

**Authors:** Sahil M. Patel, Kenneth M. Sigman, Mohannad F. Dugum

**Affiliations:** ^1^Internal Medicine, Brookwood Baptist Health, Birmingham, AL, USA; ^2^Gastro Health, Grandview Medical Center, Birmingham, AL, USA

## Abstract

Primary yolk sac tumor (YST) of the liver is an extremely rare extragonadal germ cell tumor. Here, we present a case of a young man who developed primary YST of the liver which metastasized to periductal lymph nodes causing bile duct obstruction. A 32-year-old male was referred from an outside hospital for evaluation of painless jaundice. Initial investigation showed common bile duct compression from periductal lymph nodes. Inital biopsy results were concerning for adenocarcinoma. The patient was ultimately diganosed with primary YST of the liver. He was then started on a curative-intent chemotherapy regimen of bleomycin, etoposide, and cisplatin. This case highlights the importance of keeping the primary YST of the liver on the differential diagnosis as initial staining patterns are similar to adenocarcinoma which has a very different management.

## 1. Introduction

Bile duct obstruction presents clinically with jaundice and elevated liver enzymes. Bile duct obstruction can be due to either luminal pathology such as biliary stones or strictures or extrinsic compression due to tumor or enlarged lymph nodes.

Extragonadal germ cell tumor (GCT) is a tumor that is histologically of gonadal origin despite being located outside of the gonads. The common extragonadal GCT sites are midline structures including the mediastinum, retroperitoneum, and the sacral region [1]. Primary yolk sac tumor (YST) of the liver is an extremely rare extragonadal GCT. In this case report, we highlight a case of primary YST of the liver in a young man, some peculiar features, and important learning points that can be drawn from this case.

## 2. Case Report

A 32-year-old male was referred from an outside facility for evaluation of painless jaundice. He had multiple prior hospitalizations with evidence of a distal bile duct stricture on computed tomography (CT). Endoscopic ultrasound (EUS) was performed, showing enlarged periportal and periductal lymph nodes in addition to a dilated common bile duct (CBD) secondary to the distal common bile duct (CBD) stricture. EUS-guided fine-needle biopsy (FNB) was obtained from the largest periductal lymph node. An endoscopic retrograde cholangiopancreatography (ERCP) was then performed with the placement of a plastic biliary stent to decompress the biliary tree and relieve the CBD obstruction secondary to lymphadenopathy. His serum bilirubin levels normalized 1 week later.

**Figure 1:**
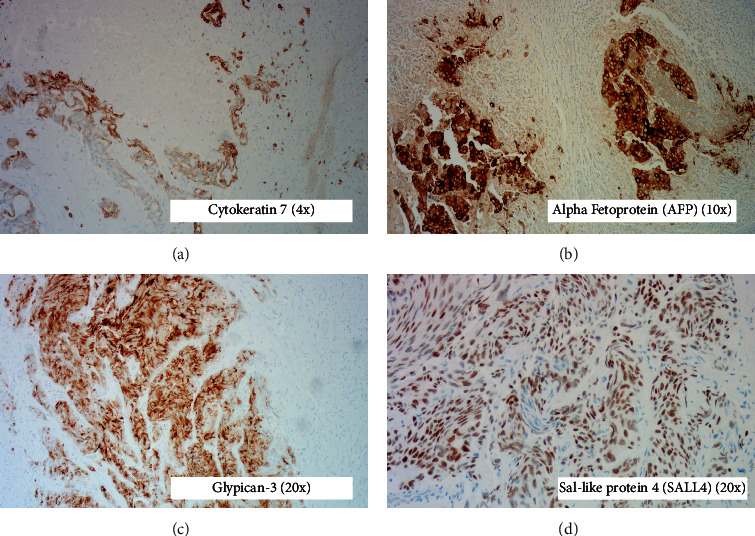
Lymph node fine needle biopsy results. (a) Positive staining for cytokeratin 7 (4x). (b) Positive staining for alpha fetoprotein (AFP) (10x). (c) Positive staining for glypican-3 (20x). (d) Positive staining for sal-like protein 4 (SALL4) (20x).

FNB results took several rounds of staining before coming to a final diagnosis. Initial staining results were significant for a positive cytokeratin 7 (Figure 1(a)) and a negative cytokeratin 17 and cytokeratin 20. The second round of staining was positive for alpha fetoprotein (AFP) (Figure 1(b)) and glypican-3 (Figure 1(c)). The final round of staining was positive for sal-like protein 4 (SALL4) (Figure 1(d)) and negative for hepatocyte paraffin-1 (HepPar-1) and arginase-1 (Arg-1). This confirmed the presence of a YST. Serum AFP was found to be elevated at >1,000 ng/mL, and serum cancer antigen 19-9 was elevated at 67.1 U/mL. A scrotal ultrasound was then performed but revealed no primary testicular mass, testicular atrophy, or scars, which confirmed the diagnosis of a primary YST of the liver.

The patient was then referred to oncology where a positron emission tomography (PET) scan was performed and showed no potential secondary sites of malignancy. He started a curative-intent chemotherapy regimen of bleomycin, etoposide, and cisplatin for a total of 4 cycles. This course was complicated by a single episode of neutropenic fever at the end of cycle 1 which led to a one-day delay in starting cycle 2. The patient ultimately required bone-marrow transplantation. He underwent ERCP 2 months after transplantation, during which the biliary stent was removed and a repeat cholangiogram confirmed resolution of the distal bile duct stricture. He is in remission as of 1 year of transplantation.

## 3. Discussion

Primary YST of the liver is a very rare extragonadal YST with only approximately 20 cases reported, the first being in 1975 by Hart et al. [2, 3]. Of note, only five of these cases were adult males [3]. The reported mortality rate is >50% [4]. This is attributed to the highly malignant nature of YSTs. Primary YST of the liver has been associated with young females and a high AFP according to Wong et al. [5]. Schiller–Duval body is pathognomonic of a YST and if identified can be a useful finding to differentiate from other malignancies.

There are three existing hypotheses for the pathogenesis of primary YST of the liver [3]. The first is aberrant migration of primordial germs which, instead of migrating towards the gonads, migrated towards a different location during embryogenesis [3]. The second hypothesis is reverse migration of the transformed germs cells where cells reach the gonads but then continue to migrate to a secondary location [3]. The third hypothesis is abnormal differentiation of somatic cells, where there were no issues with embryogenesis, but instead, the somatic cells underwent similar differentiation as germ cells in the gonads [3].

This case was unique in that the patient only became symptomatic once the tumor spread to periductal lymph nodes causing extrinsic compression of the CBD. In addition, abdominal CT and PET scan failed to find a primary mass in the liver or the testes; as a result, there was no appreciable tumor to surgically remove. Since YST is a highly malignant tumor, our hypothesis is that this primary liver tumor quickly metastasized to the periductal and periportal lymph nodes causing him to be symptomatic much earlier in his disease course. Important to note is that a routine testicular biopsy is not recommended if the scrotal ultrasound shows no testicular mass [6]. As a result, we did not elect to perform a testicular biopsy. This was further supported by the fact that there was no lumbar or para-aortic lymphadenopathy appreciated upon further investigation which could have suggested metastases of a primary testicular malignancy.

An important learning point from this case is that the FNB results took several rounds of staining before coming to the diagnosis of YST. The initial rounds of staining were suggestive of either YST, adenocarcinoma, or some rare variants of hepatocellular carcinoma. It was not until the final round of staining, showing a positive SALL4, negative HepPar-1, and negative Arg-1 which confirmed YST. Given the similar initial staining patterns between YST, adenocarcinoma, and certain variants of hepatocellular carcinoma, it is important to consider YST in the differential diagnosis. This is primarily because the management of adenocarcinoma frequently requires surgery whereas primary YST of the liver can be managed with only chemotherapy as illustrated by Whelan et al. [7]. Of note, most cases of primary YST of the liver do end up requiring surgery; however, only after the tumor burden was decreased with chemotherapy.

In conclusion, although a rare diagnosis, primary YST of the liver should be considered in the differential diagnosis of young patients with CBD obstruction secondary to extrinsic compression from periductal lymph nodes.

## Data Availability

The data that support the findings of this study are available from the corresponding author upon request.
